# Human biological variation in sesamoid bone prevalence: the curious case of the fabella

**DOI:** 10.1111/joa.13091

**Published:** 2019-10-17

**Authors:** Michael A. Berthaume, Anthony M. J. Bull

**Affiliations:** ^1^ Department of Bioengineering Imperial College London London UK

**Keywords:** bilateral/unilateral, fabella, ontogeny, prevalence, regional/global variation, sexual dimorphism

## Abstract

The fabella is a sesamoid bone located in the gastrocnemius behind the lateral femoral condyle. In humans, fabellae are 3.5 times more common today than they were 100 years ago, with prevalence rates varying between and within populations. In particular, fabellae have been assumed to be more common in Asians than non‐Asians, equally common in men and women, potentially more common in older individuals, and bilateral cases (one per knee) appear to be more common than unilateral ones. The roles of genetic and environmental factors in this phenotypic variation have been hypothesized, but not rigorously investigated. Given its clinical and evolutionary significance (i.e. being associated with several knee ailments, causing medical issues on its own, interfering with medical devices, and being less common in humans than in other mammals), it is important comprehensively to understand prevalence rate variation, and the roles of genetics and environmental factors in that variation. To address these questions, we performed a meta‐analysis on data from studies published from 1875 to 2018 to investigate possible variation in sexual dimorphic (*n* = 22 studies, 7911 knees), ontogenetic (*n* = 10 studies, 4391 knees), and global (*n* = 65 studies, 21 626 knees) fabella prevalence rates. In addition, we investigated what proportion of cases are bilateral (*n* = 37 studies, 900 individuals), and among unilateral cases (*n* = 20 studies, 204 individuals), if fabellae are more common in the left or right knee. Our results show that, today, fabellae are 2.47–2.60% more common in men than women, and prevalence rates increase ontogenetically in old age (i.e. 70 years old), implying that fabellae can ossify early (i.e. 12 years old) or late in life. Approximately 72.94% of cases are bilateral, and among unilateral ones, fabellae are equally common in right and left knees. There is marked regional variation in fabella prevalence rates, with rates being highest in Asia, followed by Oceania, South America, Europe, Middle East, and North America, and lowest in Africa. Worldwide, an average of 36.80% of knees has ossified fabellae detectable by dissection. These results imply that, while the ability to form a fabella may be genetically controlled, the mechanisms that trigger fabella ossification may be environmentally controlled. What these environmental factors are, can only be speculated.

## Graphical abstract

The fabella, a sesamoid bone in the knee, is ~3.5× more common today than 100 years ago. We performed meta‐analyses on prevalence rate studies from 1875–2019, and found (i) fabellae are more common in men, older individuals, and Asian/Oceania populations than women, younger individuals, and North American/African populations (ii) 72.94% of cases are bilateral, and (iii) among unilateral cases, there is anti‐symmetry. This supports the roles both genetics and environment in the fabella phenotype.

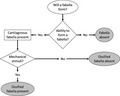

## Introduction

The fabella, Latin for ‘little bean’, is a sesamoid bone located behind the lateral femoral condyle in the lateral head of the gastrocnemius. Although common in non‐hominoid mammals, prevalence rates range from 3 to 87% in humans (Sarin et al. 1999; Silva et al. 2010; Zeng et al. 2012; Jin et al. 2017). A range of genetic and environmental explanations have been proposed to explain this variation, but few studies consider more than one population for statistical analyses. Understanding the influence of genetics and environment on variation in prevalence rates using a multi‐population, global approach, is of evolutionary and medical significance. This is particularly important today, as fabellae are ~ 3.5 times more common today than 100 years ago, possibly because better nutrition has made people taller/heavier, meaning they presumably have longer tibiae and moment arms about the knee, and larger gastrocnemii, which together produce more mechanical stimuli that may provide signalling for fabella formation/ossification (Berthaume et al. 2019).

Fabella presence is correlated with some anatomical structures in the knee, and affects how loads are transferred from the gastrocnemius to the femur, tibia, and fibula. When present, the fabella is often accompanied by the fabellofibular ligament, which connects the distal insertion of the fabella to the fibular head (Minowa et al. 2004; Piyawinijwong et al. 2012; Driessen et al. 2014; Hauser et al. 2015; Kurtoğlu et al. 2015). In some rare cases, the fabella serves as an additional origin for a muscle bundle of the popliteal muscle (Bejjani & Jahss, 1985; Duc et al. 2004). This should not be confused with the cyamella, a sesamoid bone located in the proximal tendon of the popliteus muscle, located distolaterally to the fabella (Akansel et al. 2006). Finally, it has been suggested by some researchers that the arthrodia (gliding) joint between the fabella and femur creates a morphologically unique fourth compartment of the knee (Lencina, 2007; Zeng et al. 2012; Ehara, 2014). This is supported by the morphological changes that sometimes are present in the posterior surface of the lateral femoral condyle (Berthaume et al. 2019).

Sesamoid bones have a long evolutionary history dating back to the Jurassic period, approximately 150–200 million years ago (see Sarin et al. 1999 for discussion). They are hypothesized to develop within tendons to reduce tendon damage in areas that experience high tensile strain and hydrostatic compressive mechanical stresses (Sarin et al. 1999). In humans, the fabella has been suggested to serve as a stabilizer of the posterolateral aspect of the knee (Phukubye & Oyedele, 2011; Tabira et al. 2012) and, in mammals, it may increase mechanical advantage of the gastrocnemius (Sarin et al. 1999). Coincident development of the fabella and other sesamoid bones suggests that the ability of sesamoid bones to form may be genetically controlled (Sarin et al. 1999). Genetic control of the fabella is supported by the high prevalence of bilateral cases (one fabella per knee), ranging from 50 to 97% (Chung, 1934; Kojima, 1958; Houghton‐Allen, 2001; Phukubye & Oyedele, 2011; Piyawinijwong et al. 2012; Egerci et al. 2017).

The role of genetic and developmental pathways in sesamoid bone development was recently investigated in a mouse model (Eyal et al. 2015, 2019). The patella, fabella, and digit sesamoids were found to originate from Sox9^+^/Scx^+^ progenitors, under the regulation of transforming growth factor β (TGFβ), independent of muscular mechanical stimuli. BMP2 was found to regulate sesamoid growth, but the differentiation of the fabella and digit sesamoids were regulated redundantly by BMP4 and BMP2, implying the ability to form sesamoids is genetically controlled, and not all sesamoids have the same developmental pathways (Eyal et al. 2019).

The role of genetic factors in fabella development is further supported by dissections of 15‐ to 18‐week‐old foetuses, which showed cartilaginous fabellae in 9/12 of the knees examined (*n* = 8 individuals), which likely experience little/no mechanical stimuli in the knee (Jin et al. 2017). Dissection of a 4.3‐cm human fetus led to the hypothesis that the fabella originates as a cartilaginous fragment on the fibular head that is detached by the fabellofibular ligament during growth (Fürst, 1903; Fabbriciani & Oransky, 1992), similar to how the mouse patella develops as a bony process on the femur, which is later separated and superficially embedded in the quadriceps tendon (Eyal et al. 2015). If true, this could suggest that the temporal increase in prevalence rates reflect changes in utero and/or an increase in ossified, but not cartilaginous, fabellae. The latter is plausible, as many studies often ignore less dense, ossified fabellae.

The anatomical effects of the presence of the fabella are of interest to orthopaedic surgeons. In cases of total knee arthroplasty (TKA), the fabella can cause postsurgical complications by snapping over the replacement knee joint (Larson & Becker, 1993; Erichsen, 1997; Segal et al. 2004; Theodorou et al. 2005; Hou, 2016; Kwee et al. 2016; Okano et al. 2016). This may be because the replacement knee does not reflect the morphology of the original knee. When fabellae are present, the posterior section of the lateral femoral condyle can have an articulating groove that stabilizes the fabella (Chew et al. 2014). This is absent in replacement knees, which can cause the fabella painfully to ‘snap’ over the replacement condyle, possibly because of medio‐lateral fabella instability and/or increased tension in the tendon. The reason for this pain unknown. A clinical trial investigated the benefits of removing fabellae, when present, at the time of TKA. While those with fabellae removed never suffered from the painful snapping, those with fabellae left in place occasionally did, and consequently required a fabellectomy. This work resulted in the recommendation to remove fabellae during TKA surgery if present (Hou, 2016).

In addition, the fabella is found in association with several conditions, disorders, and diseases, including common peroneal neuropathy (Mangieri, 1973; Patel et al. 2013; Cesmebasi et al. 2016), chondromalacia (Goldenberg & Wild, 1952; Grisolia & Bartels, 1959; Robertson et al. 2004), osteoarthritis (Hagihara et al. 1994), nerve palsy (Itoman et al. 1976; Takebe & Hirohata, 1981; Kubota et al. 1986; Tabira et al. 2012; Décard et al. 2017), popliteal artery entrapment syndrome (Ando et al. 2017), and rheumatoid arthritis (Uchino et al. 1992). The fabella itself can cause medical issues through dislocation (Frey et al. 1987; Franceschi et al. 2007), fracture (Sagel, 1932; Levowitz & Kletschka, 1955; Ikeuchi & Nagatsuka, 1970; Dashefsky, 1977; Woo, 1988; Marks et al. 1998; Theodorou et al. 2005; Tang et al. 2010; Heideman et al. 2011; Barreto et al. 2012; Cherrad et al. 2015; Kwee et al. 2016; Zhou et al. 2017), and fabella syndrome (Weiner et al. 1977; Weiner & Macnab, 1982; Erichsen, 1997; Zipple et al. 2003; Segal et al. 2004; Dannawi et al. 2010; Seol et al. 2016; Kim et al. 2018), which is posterolateral knee pain associated with the presence of a fabella (Driessen et al. 2014).

Perhaps the most interesting association between the fabella and medical issues is that between fabella presence and osteoarthritis in the knee. If an individual has osteoarthritis in their knee, they are twice as likely to have a fabella in that knee compared with an age‐matched cohort with radiographically normal knees (Pritchett, 1984; Hagihara et al. 1993). It is unclear whether the fabella is causative of – or contributing to – osteoarthritis, arising from osteoarthritis, or if fabella presence and osteoarthritis are symptoms of another condition.

In prevalence rate studies, fabella presence is often determined through surgeries/dissection (Phukubye & Oyedele, 2011; Agathangelidis et al. 2016), X‐rays, (Pancoast, 1909), computed tomography (CT) scans (Hauser et al. 2015) or magnetic resonance imaging (MRI) scans (Hedderwick et al. 2017). Although ultrasound (Sekiya et al. 2002) and PET‐CTs (Usmani et al. 2017) can detect fabellae, it has not been used for prevalence rate studies. The method of data collection affects the reported prevalence rate (Berthaume et al. 2019). For example, small structures in the posterolateral aspect of the knee are easy to miss if the knee is positioned incorrectly (Yu et al. 1996; Ehara, 2014), decreasing the documented prevalence rate. Additionally, cartilaginous and less dense, ossified fabellae detectable by dissection and MRI (Phukubye & Oyedele, 2011; Jin et al. 2017) are not always detectable by X‐ray or CT scan. For example, Zeng et al. (2012) reported 57.9% of the cartilaginous fabella were not visible on radiographs. This makes it difficult to compare prevalence rates between studies, as not all studies differentiate between cartilaginous/less dense and denser, ossified fabellae, or state whether cartilaginous fabellae were recorded.

Variation in human fabella sexual dimorphic, ontogenetic, bilateral prevalence and global prevalence rates have been investigated since 1875 (Gruber, 1875). Prevalence rates range from 4.3 to 52.8% (Frey, 1913 cited by Hessen 1946 and Ghimire et al. 2017)) in males and 3.3–50% (Sugiyama, 1914 cited by Chung, 1934 and Kaneko, 1966) in females, although sexual dimorphic differences are often unreported because of small or unbalanced samples (e.g. Yano, 1928).

Ontogenetically, cartilaginous fabellae have been found in fetuses as young as 15 weeks (Jin et al. 2017) and ossified fabellae in individuals as young as 12 years (Pancoast, 1909; Ehara, 2014). The age of fabella ossification is unknown. Berthaume et al. (2019) investigated the relationship between fabella prevalence and age at death in a population of adult Koreans (21–60 years old, median age 55 years) but found none, implying ossification may occur before adulthood. One study reported a correlation between fabella prevalence and age in adults, with fabellae being more common in individuals > 50 years old at the time of knee imaging (Kato et al. 2012), implying that ossification could occur quite late in life. However, Berthaume et al. (2019) found no association between fabella prevalence and individuals younger/older than 50 years, and thus could not support the conclusions of Kato et al. (2012). Several studies have qualitatively assessed the relationship between binned age categories and prevalence rates, but small sample sizes per age group prevent definitive results from being drawn. A problem with studies investigating fabella ontogeny is the investigation of fabella presence/absence at distinct ages (generally, age of death or age of medical knee imaging). The use of cross‐sectional data prevents determination of age of fabella formation/ossification. Together, these studies imply fabellae may begin formation as early as *in utero*, and may ossify at any time during juvenility/adulthood.

Bilateral fabellae (one per knee) are more common than unilateral fabellae (one per individual), and within unilateral cases, fabellae appear equally likely to be found in either right or left knees (Berthaume et al. 2019). Globally, fabellae are understood to be more common in Asian populations, with prevalence rates reaching 87% in Japan (Zeng et al. 2012), than non‐Asian populations, which generally have prevalence rates of 10–30% (Duncan & Dahm, 2003). One study compared prevalence rates among three human populations and found regional variation in prevalence rates (Miaśkiewicz & Partyka, 1984).

Here, we perform worldwide meta‐analyses of fabella prevalence rates to address the following questions:

1. Are there sexually dimorphic differences in fabella prevalence rates?
2. How do prevalence rates change with ontogeny?
3. Are bilateral or unilateral cases more common? Among unilateral cases, are fabellae more common in right or left knees?
4. What is the global prevalence rate of the fabella? How do these rates vary in different regions of the world?

As no previous studies have reported on sexually dimorphic differences, we hypothesize there will be no sexually dimorphic differences in our analyses. Some studies have suggested ossified fabellae are more common in older individuals (Chung, 1934), and as such we hypothesize prevalence rates will increase with ontogeny. Bilateral fabellae are reportedly much more common than unilateral fabellae, and within unilateral cases, authors have not reported on fabella being more/less common in one knee or the other (Berthaume et al. 2019). As such, we hypothesize bilateral fabellae will be more common than unilateral ones, and in unilateral cases, fabellae will be equally common in right and left knees. Finally, authors have suggested fabellae are more common in some populations (e.g. Asians) and less common in others (e.g. Europeans; Miaśkiewicz & Partyka, 1984). As such, we hypothesize there will be variation in fabella prevalence rates between populations.

## Materials and methods

A previously published systematic review identified 66 studies from 1875 to 2018 on google.scholar.co.uk that reported on fabella prevalence rates, using the following search terms: fabella sesamoid, fabellae sesamoid, fabella knee, fabellae knee, cyamella, fabella incidence rate, fabellae incidence rate, fabella prevalence rate, and fabellae prevalence rate. Studies were written in seven languages (English, German, French, Spanish, Italian, Japanese, and Chinese) and translated either with native speakers or using google translate. One additional study (Silva et al. 2010), excluded in Berthaume et al. (2019) because it was an outlier for that analysis, was not an outlier for the bilateral/unilateral analysis and was included here. It was excluded from the regional and global analyses, where it was still an outlier. Although samples sometimes represented random, asymptomatic individuals, this was not always the case. For example, some studies were performed on cadaveric samples in teaching labs, and some were based on hospital records. In addition, some earlier studies were skewed to have a larger male sample.

Data on sexual dimorphism, ontogeny, and bilateral/unilateral fabella presence were extracted. The year the study was published, method used to collect the data (dissection, X‐ray, MRI, or CT scan), and country of the first author were recorded. Data on cartilaginous fabellae are occasionally reported (Parsons & Keith, 1897; Chung, 1934; Tabira et al. 2012; Corvalan et al. 2018; Tatagari et al. 2018). Here, cartilaginous fabellae were ignored when possible, as cartilaginous fabellae are not dense enough to be detected by X‐ray, and may or may not have been included in dissection studies. Country was defined either by sample provenance (if specified) or by country of the first author; often, all authors came from the same country.

### Inclusion criteria

Prevalence rates are reported either per individual or per knee. Per knee prevalence rates are more common, as in X‐ray studies both knees are not always imaged, and dissection studies can have isolated knees. As such, we only included studies with knee prevalence rates or studies in which individual prevalence rates could be transformed to knee prevalence rates. Only individuals who had both knees examined could be included for the bilateral/unilateral analysis. One additional study was published on fabella prevalence after the systematic review (Berthaume et al. 2019). This study was excluded from this analysis, as it reported on individual, and not knee, prevalence rates, and it was not possible to transform the individual prevalence rates to knee rates (Pop et al. 2018).

As in Berthaume et al. (2019), studies were excluded if their sample consisted of fewer than 12 knees, and if studies reported on cartilaginous and ossified fabellae, separately, the data on cartilaginous fabellae were not used to calculate prevalence rates. Additionally, if there were identifiable biases, such as only including people with fabellae, they were excluded from the study.

### Data manipulation

There were no obvious outliers for any of the analyses in this study. If studies used populations from more than one country, and the country of origin was specified (Miaśkiewicz & Partyka, 1984), each country was treated as a separate study. Similarly, if studies used two different methodologies for data collection (Lencina, 2007), they were treated as separate studies. The methodology used for data collection could not be determined for five studies: in these cases, we used the imputed values published in Berthaume et al. (2019).

Most studies reported on ontogenetic results by binning age in years. The most common bins were 0–10, 11–20, 21–30, … 71–80, 81+, and thus studies with these bins, or studies for which we had access to the raw data, were used for this analysis. Given the paucity of data, if studies reported bins that differed by 1 year (e.g. 10–19 instead of 11–20), the data were also included here. Data for Gruber (1875) were taken from Rothe (1927), where data were binned in 10‐year intervals but the lowest bin was 10–30 (*n* = 230) and the highest bin 60+ (*n* = 28). As the 10–30 bin is equally likely to be included in the 11–20 and 21–30 bins, and 230 individuals represents a large percentage of the 11–20 age bin (*n* = 310), potentially skewing the results, the data were ignored. As 28 individuals represent a small percentage of the 61–70 bin, and, based on age distributions from other studies, there is likely to be a larger proportion of 61–70 year‐old individuals than 71+ year‐old individuals, they were included in the 61–70 bin. Similarly, Hessen's (1946) last bin was 71+ (*n* = 58), and this was included in our 71–80 bin.

One study with the proper bins plotted prevalence rates per age group in terms of percentages of individuals with fabellae (Ghimire et al. 2017). We contacted the corresponding author, and were supplied with the data used to calculate those percentages, which were necessary for our analyses (now available on http://www.researchgate.net). Another study plotted the number of individuals with and without fabellae per age group by sex, and provided totals for the number of males and females with and without fabellae (Lungmuss, 1954). We did our best to estimate sample sizes from the plot, but our estimates for the total number of males and females with (n_male_ = 160, n_female_ = 44) and without (n_male_ = 610, n_female_ = 202) fabellae did not match their totals (with fabellae, n_male_ = 153, n_female_ = 39; without fabellae, n_male_ = 615, n_female_ = 193). As our totals were close the reported ones, we used our numbers in these analyses (Lungmuss, 1954).

As several countries were represented by only one study, countries were coded by region (Africa, Asia, Europe, Middle East, North America, Oceania, and South America). In addition, as only one study utilized CT scans, and CT scans use the same detection methodology for data collection as X‐rays, this study was coded as X‐ray, giving three possible levels for methodology (dissection, MRI, and X‐ray). MRI data were not coded as dissection, as it is possible to miss anatomical structures in the posterolateral component of the knee during MRI that are visible during dissection (Munshi et al. 2003; Hedderwick et al. 2017).

### Statistical models

Bayesian binomial mixed‐effects linear models were used to investigate sexual dimorphic, ontogenetic, bilateral, and global prevalence rates. Previous studies indicate that the effects of region, method for data collection, and year in which the study was conducted could all potentially affect prevalence rate distribution. As such, models were run taking into account the random effects of region and method through random intercepts, and fixed effect of year. If one or more of the effects appeared to be statistically insignificant, additional models were run with these parameters removed and Watanabe–Akaike information criteria (WAIC) was used to compare the models.

For the ontogenetic analysis, age ranges were treated as factors, and not integer/numeric, allowing for prevalence rates to be estimated for each age range, independently. For the regional analysis, two models were run, both including all parameters. The first was a random slope model, including the random slopes of country and method, as it is possible the relationship between our explanatory and response variables differs within each group, and the second was a random intercept model.

All statistical analyses were run in R and rstudio using the rethinking, ggplot2, and gridextra packages (Wickham, 2009; Team R, 2015; McElreath, 2016; Auguie, 2017; R Core Team, 2018). All raw data and posterior distributions for the parameters are available in Supporting Information Appendix S1).

## Results

**Figure 1:**
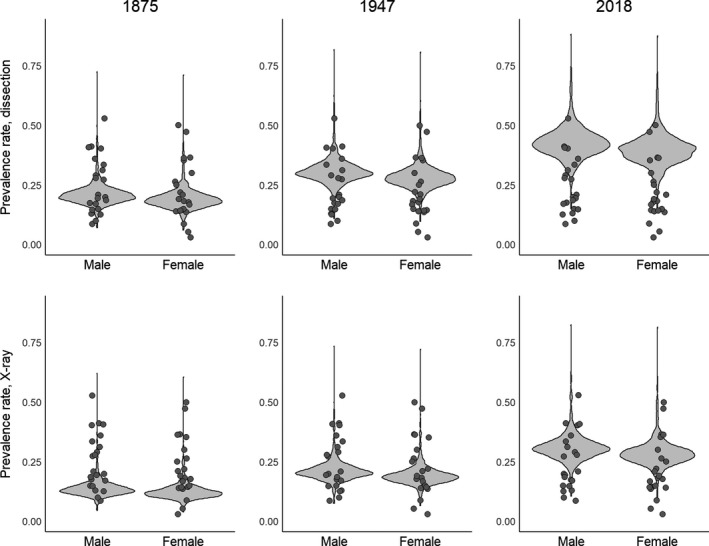
Sexual dimorphic effects of prevalence rates (*n* = 22 studies, 8066 knees). Prevalence rates increase with time, and are higher for dissection‐based studies than for X‐ray‐based ones, possibly because the dissection‐based studies include some cartilaginous/less dense, ossified fabellae. Raw data are scattered on top of the violin plot, which was constructed by resampling the posterior distribution and creating a hypothetical dataset of prevalence rates.

**Figure 2:**
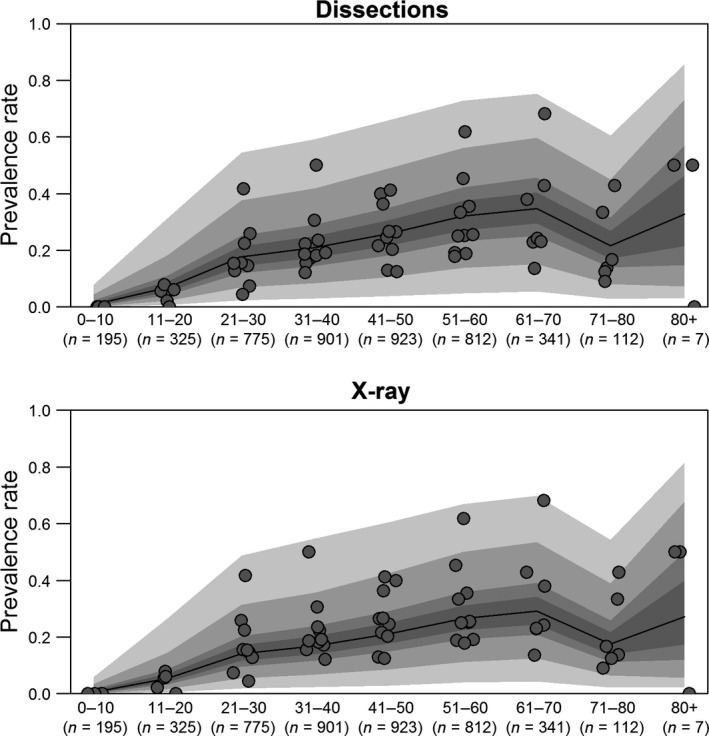
Ontogenetic effects of prevalence rates (*n* = 10 studies, *n* = 4391 knees). Prevalence rates increase with age, and are higher for dissection‐based studies than for X‐ray‐based ones, possibly because the dissection‐based datasets studies include some cartilaginous/less dense ossified fabellae. The median prevalence rate is represented by the black solid line, and is framed by the 50%, 75%, 95%, and 99% confidence intervals. Raw data are plotted as a scatter. Sample sizes are given under the age range in terms of number of knees per age range.

**Figure 3:**
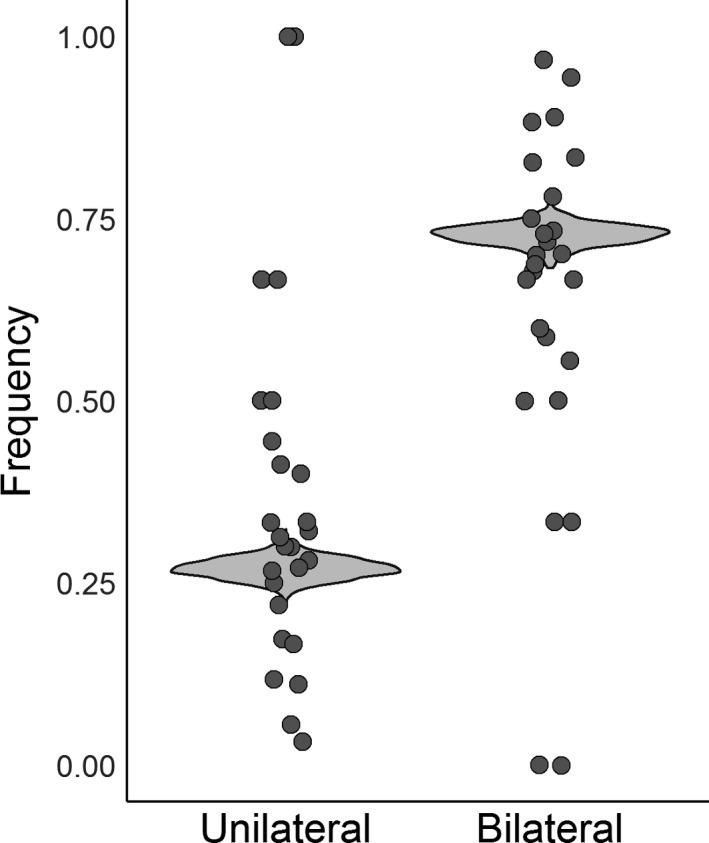
Frequency of unilateral and bilateral cases (*n* = 37 studies, 900 individuals). Raw data are scattered on top of the violin plot, which was constructed by resampling the posterior distribution and creating a hypothetical dataset of prevalence rates. The median frequency of unilateral and bilateral cases is 27.0% and 72.94%, respectively.

**Figure 4:**
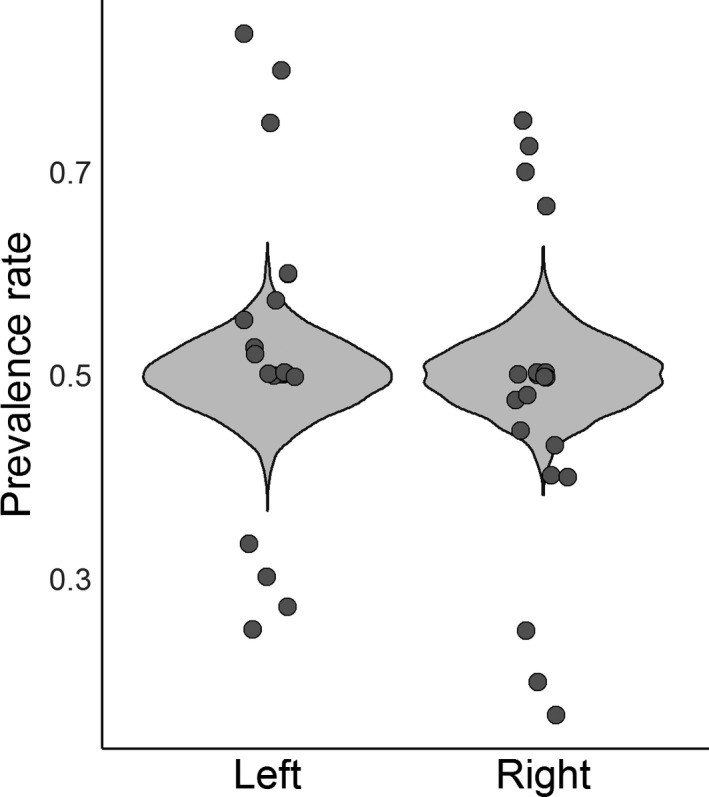
Within unilateral cases (*n* = 20 studies, 204 individuals), fabellae are equally likely to be found in right and left knees (*P* = 0.9992). Raw data are scattered on top of the violin plot, which was constructed by resampling the posterior distribution and creating a hypothetical dataset of prevalence rates.

**Figure 5:**
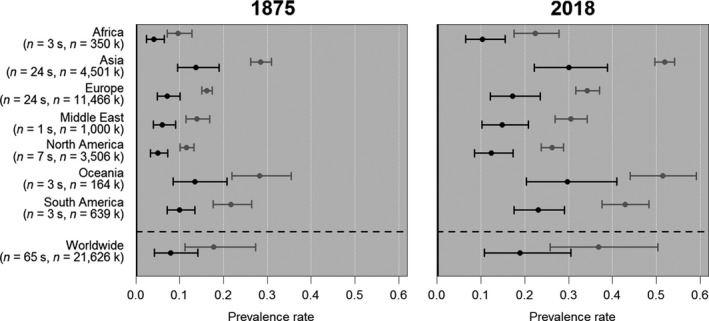
Fabella prevalence rates for 1875 and 2018. Black and grey lines indicate prevalence rates using X‐rays and dissections, respectively. Prevalence rates are given for each region and worldwide. Worldwide estimates were calculated using average effect of region. Datapoints are medians and error bars are 95% confidence intervals. Sample sizes are given in number of studies (s) and knees (k).

Median prevalence rates, with their 50%, 75%, 95%, and 99% confidence intervals (depicted in Figs 1, 2, 3, 4, 5) are available in Supporting Information Appendix S2.

### Sexual dimorphism

There were 22 studies from 1875 to 2018 that contained sex‐specific data, providing a sample of 7911 knees (*n* = 5161 males, *n* = 2750 females) representing three regions (Asia, Europe, and South America) and two methods of data collection (dissection and X‐ray; Table 1). Four models were run to investigate sexual dimorphism; the best model accounted for all random and fixed effects (Supporting Information Appendix S3, weight = 1). Posterior distributions for the parameters in this model can be found in Supporting Information Appendix S4. Prevalence rates were higher in men than women (*P* = 0.048), and prevalence rates increased through time for both men and women (*P* < 0.0001). Prevalence rates derived from dissections are higher than those derived from X‐rays.

**Table 1:** Data used for the sexual dimorphic analysis. Source is the source from which the data were gathered, *n* = sample size, F = number of fabellae. CT scan was coded as X‐ray for statistical analysis. Data from Ghimire et al. (2017) were provided to the authors directly.

Author	Year	Source	Method	Region	Sex	Female		Male	
						*n*	F	*n*	F
Gruber	1875	Gruber, (1875)	Dissection	Europe	F	100	22	840	143
Ost	1877	Ost, (1877)	Dissection	Europe	F	10	3	20	2
Pfitzner and Schwalbe	1892	Pfitzner & Schwalbe (1892)	Dissection	Europe	F	93	5	198	25
Parsons and Keith	1897	Parsons & Keith, (1897)	Dissection	Europe	F	84	21	157	49
Frey	1913	Frey, (1913)	Dissection	Europe	F	33	1	80	14
Sugiyama	1914	Hessen, (1946)	Dissection	Asia	F	22	8	53	28
Hanamuro	1927	Hessen, (1946)	X‐ray	Asia	F	200	42	200	72
Rothe	1927	Rothe, (1927)	X‐ray	Europe	F	198	27	402	59
Yano	1928	Yano, (1928)	Dissection	Asia	F	14	2	151	42
Ooi (Oi?)	1930	Kaneko, (1966)	Dissection	Asia	F	53	14	27	11
Sonntag	1930	Hessen, (1946)	X‐ray	Europe	F	242	36	448	83
Mikami	1932	Hessen, (1946)	X‐ray	Asia	F	195	17	315	61
Chung	1934	Chung, (1934)	Dissection	Asia	F	82	15	266	89
Kitahara	1935	Kitahara, (1935)	X‐ray	Asia	F	100	14	100	13
Hessen	1946	Hessen, (1946)	X‐ray	Europe	F	474	84	468	70
Lungmuss	1954	Lungmuss, (1954)	X‐ray	Europe	F	232	39	768	153
Kojima	1958	(Kojima, (1958)	Dissection	Asia	F	52	19	100	29
Kaneko	1966	Kaneko, (1966)	Dissection	Asia	F	26	13	124	50
Ghimire, et al.,	2017	(Ghimire et al. (2017)	X‐ray	Asia	F	97	14	58	5
Lencina	2007	Lencina, (2007)	X‐ray	South America	F	21	4	196	41
Ortega and Olave	2018	Ortega & Olave, (2018)	X‐ray	South America	F	312	110	88	24
Berthaume et al.,	2019	Berthaume et al. (2019)	CT scans	Asia	F	110	52	102	42

Assuming average effect of regions, the median prevalence rates in 2018 for dissections were 42.27% and 39.67% (95% confidence intervals: 30.62–57.32% and 27.86–55.59%), and for X‐rays 30.44% and 27.97% (95% CI 21.60–44.50% and 19.30–43.04%) for men and women, respectively (Fig. 1). Prevalence rates for 1875 and 1947, and additional confidence intervals (99%, 75%, and 50%) can be found in Appendix S2.

### Ontogeny

There were 10 studies that met our criteria from 1875 to 2018, providing a total of 4391 knees across all age ranges, representing three regions (Asia, Europe, and North America) and two methods of data collection (dissection and X‐ray; Table 2). Only five studies provided sex‐specific age information: had sex been included in this analysis, our sample size would have nearly been halved. As such, the effects of sex were ignored. The same four models were run to investigate ontogeny; the best model included region and method, but not year (Appendix S3, weight = 0.53). This model only performed marginally better than the model that included year. Posterior distributions for the parameters in this model can be found in Appendix S4. Fabellae were generally more common in older individuals than in younger ones (Fig. 2). The apparent decrease in prevalence rates in the 71–80 and 80+ bins are likely due to small sample sizes (*n* = 112 and 7, respectively). As might be expected, prevalence rates are higher in 2018 than in 1875, and in data derived from dissections.

**Table 2:** Data used for the ontogenetic analysis. Numbers in the top row signify age bins. C in the Sex column signifies combined males and females. *n* = sample, F = number of fabellae. CT scan was coded as X‐ray for statistical analysis. Data from Gruber (1875) for their 10–30 year bin were omitted, and the data for the 60+ bin were placed in our 61–70 bin. From Hessen (1946), data from their 70+ bin were placed in our 71–80 bin. Data from Ghimire et al. (2017) were provided to the authors directly.

Author	Year	Source	Method	Region	Sex	0–10		11–20		21–30		31–40		41–50		51–60		61–70		71–80		81–90	
						*n*	F	*n*	F	*n*	F	*n*	F	*n*	F	*n*	F	*n*	F	*n*	F	*n*	F
Gruber	1875	Gruber, (1875)	Dissection	Europe	C	NA	NA	NA	NA	NA	NA	93	16	68	18	45	16	28	12	NA	NA	NA	NA
Yano	1928	Yano, (1928)	Dissection	Asia	M	NA	NA	NA	NA	37	6	39	9	32	13	28	7	13	9	2	0	NA	NA
Yano	1928		Dissection	Asia	F	NA	NA	NA	NA	2	0	8	0	2	1	NA	NA	NA	NA	2	0	NA	NA
Yano	1928		Dissection	Asia	C	NA	NA	NA	NA	39	6	47	9	34	14	28	7	NA	NA	NA	NA	NA	NA
Sonntag	1930	(Sonntag, (1930)	X‐ray	Europe	C	16	0	98	6	185	29	128	24	128	26	94	24	33	8	6	1	2	1
Chung	1934	Chung, (1934)	Dissection	Asia	M	18	0	12	1	46	13	67	21	59	15	36	24	20	13	8	2	NA	NA
Chung	1934		Dissection	Asia	F	32	0	6	0	12	2	8	2	6	1	6	2	2	2	6	4	4	2
Chung	1934		Dissection	Asia	C	50	0	18	1	58	15	75	23	65	16	42	26	22	15	14	6	4	2
Kitahara	1935	Kitahara, (1935)	X‐ray	Asia	M	NA	NA	6	1	22	3	13	2	7	2	2	1	NA	NA	NA	NA	NA	NA
Kitahara	1935		X‐ray	Asia	F	NA	NA	14	1	19	3	9	2	4	2	1	0	NA	NA	NA	NA	NA	NA
Kitahara	1935		X‐ray	Asia	C	NA	NA	NA	NA	41	6	22	4	11	4	3	1	NA	NA	NA	NA	NA	NA
Sutro	1935	Sutro et al. (1935)	X‐ray	North America	C	71	0	92	2	102	13	123	15	147	19	117	22	39	9	8	1	1	0
Hessen	1946	(Hessen, (1946)	X‐ray	Europe	C	58	0	102	8	174	13	126	28	150	40	156	30	118	27	58	8	NA	NA
Lungmuss	1954	Lungmuss, (1954)	X‐ray	Europe	M	NA	NA	66	2	96	21	211	30	215	52	112	26	59	24	11	5	NA	NA
Lungmuss	1954		X‐ray	Europe	F	NA	NA	65	5	46	11	51	11	25	0	35	11	20	6	4	0	NA	NA
Lungmuss	1954		X‐ray	Europe	C	NA	NA	NA	NA	142	32	262	41	240	52	147	37	79	30	15	5	NA	NA
Ghimire et al.,	2017	Ghimire et al. (2017)	X‐ray	Asia	C	NA	NA	15	15	25	22	35	17	45	40	55	28	65	22	75	11	NA	NA
Berthaume et al.,	2019	Berthaume et al. (2019)	CT scan	Asia	M	NA	NA	NA	NA	8	5	4	2	22	8	68	27	NA	NA	NA	NA	NA	NA
Berthaume et al.,	2019		CT scan	Asia	F	NA	NA	NA	NA	4	0	4	2	18	8	84	42	NA	NA	NA	NA	NA	NA
Berthaume et al.,	2019		CT scan	Asia	C	NA	NA	NA	NA	12	5	8	4	40	16	152	69	NA	NA	NA	NA	NA	NA

Assuming average effects of region, the median prevalence for 21‐ to 30‐year‐olds in 2018 is 29.9% and 20.9% when data are collected with dissections and X‐rays, respectively. The median prevalence rates in 2018 for the 31–40, 41–50, 51–60, and 61–70 year‐old bins were 20.94%, 25.87%, 32.14%, 34.72%, and 21.59% for dissections, and 16.94%, 21.21% 26.62%, 29.09%, and 17.51% for X‐rays, respectively. Median prevalence rates and confidence intervals can be found in Appendix S2.

### Bilateral/unilateral

In all, 37 studies contained individuals in which both knees were examined from 1875 to 2018, providing a sample of 900 individuals, representing all regions and two methods of data collection (dissection and X‐ray; Table 3). Four models were run to calculate the frequency of unilateral and bilateral cases, independently, meaning the total percentage of cases may not add up to exactly 100%. The best model excluded the effects of region, method, and year (Appendix S3, weight = 0.63). Posterior distributions for the parameters in this model can be found in Appendix S4. Bilateral cases were significantly more common than unilateral cases (*P* < 2.778e‐05; Fig. 3). The median chance of having two fabellae is 72.94% (95% CI 69.82–75.73%), and one fabella 26.99% (95% CI 24.26–29.91%). Additional confidence intervals are provided in Appendix S2.

**Table 3:** Data used for bilateral vs. unilateral analysis. CT scan was coded as X‐ray for statistical analysis.

Author	Year	Source	Method	Region	Number bilateral cases	Number unilateral cases
Gruber	1875	Gruber (1875)	Dissection	Europe	69	27
Pfitzner and Schwalbe	1892	Pfitzner & Schwalbe (1892)	Dissection	Europe	11	5
Pancoast	1909	Pancoast (1909)	X‐ray	North America	14	6
Frey	1913	Frey (1913)	Dissection	Europe	5	4
Sugiyama	1914	Hessen (1946)	Dissection	Asia	16	2
Hanamuro	1927	Hessen (1946)	X‐ray	Asia	47	20
Rothe	1927	Rothe (1927)	X‐ray	Europe	0	2
Yano	1928	Yano (1928)	Dissection	Asia	27	10
Ooi (Oi?)	1930	Kaneko (1966)	Dissection	Asia	3	6
Siina	1931	Hessen (1946)	X‐ray	Europe	1	2
Chung	1934	Chung (1934)	Dissection	Asia	61	2
Kitahara	1935	Kitahara (1935)	X‐ray	Asia	10	7
Sutro et al.	1935	(Sutro et al. (1935)	X‐ray	North America	15	2
Kojima	1958	Kojima (1958)	Dissection	Asia	18	12
Falk	1963	Falk (1963)	X‐ray	North America	39	11
Kaneko	1966	Kaneko (1966)	Dissection	Asia	25	5
Johnson & Brogdon	1982	Johnson & Brogdon (1982)	X‐ray	North America	24	5
Lencina	2007	(Lencina, 2007)	Dissection	South America	1	1
Silva et al.	2010	Silva et al. (2010)	Dissection	South America	0	2
Phukubye & Oyedele	2011	Phukubye & Oyedele (2011)	Dissection	Africa	6	6
Tabira et al.	2012	Tabira et al. (2012)	Dissection	Asia	34	2
Hauser et al.	2015	Hauser et al. (2015)	Dissection	Europe	45	15
Egerci et al.	2017	Egerci et al. (2017)	X‐ray	Middle East	76	38
Ortega & Olave	2018	Ortega & Olave (2018)	X‐ray	South America	50	25
Tatagari et al.	2018	Tatagari et al. (2018)	Dissection	North America	22	8
Berthaume et al.	2019	Berthaume et al. (2019)	CT scan	Asia	38	18

Among unilateral cases, we investigated whether fabellae were more common in the left or right knee. There were 20 studies with unilateral cases and side‐specific information from 1875 to 2018, providing a sample of 204 individuals, representing all regions except Oceania and two methods of data collection (dissection and X‐ray; Table 4). Four models were run to examine whether fabellae were more often present in one knee than the other. The best model excluded the effects of region, method, and year (Appendix S3, weight = 0.60). Fabellae were no more common in the right or left knee (*P* = 0.9992; Fig. 4).

**Table 4:** Data used for side preference analysis. CT scan was coded as X‐ray for statistical analysis.

Author	Year	Source	Method	Region	Number of knees	Fabellae in right knee	Fabellae in left knee
Gruber	1875	Gruber (1875)	Dissection	Europe	27	18	9
Pfitzner and Schwalbe	1892	Pfitzner & Schwalbe (1892)	Dissection	Europe	5	2	3
Frey	1913	Frey (1913)	Dissection	Europe	4	3	1
Sugiyama	1914	Hessen (1946)	Dissection	Asia	2	1	1
Hanamuro	1927	Hessen (1946)	X‐ray	Asia	20	14	6
Yano	1928	Yano (1928)	Dissection	Asia	10	2	8
Ooi (Oi?)	1930	Kaneko (1966)	Dissection	Asia	6	1	5
Siina	1931	Hessen (1946)	X‐ray	Europe	2	1	1
Chung	1934	Chung (1934)	Dissection	Asia	2	1	1
Kitahara	1935	Kitahara (1935)	X‐ray	Asia	7	3	4
Sutro et al.,	1935	Sutro et al. (1935)	X‐ray	North America	2	1	1
Kojima	1958	Kojima (1958)	Dissection	Asia	12	3	9
Falk	1963	Falk (1963)	X‐ray	North America	11	8	3
Kaneko	1966	Kaneko (1966)	Dissection	Asia	5	2	3
Lencina	2007	Lencina (2007)	Dissection	South America	0	0	0
Silva et al.	2010	Silva et al. (2010)	Dissection	South America	2	1	1
Phukubye & Oyedele	2011	Phukubye & Oyedele (2011)	Dissection	Africa	6	3	3
Egerci et al.	2017	Egerci et al. (2017)	X‐ray	Middle East	38	18	20
Ortega and Olave	2018	Ortega & Olave (2018)	X‐ray	South America	25	12	13
Berthaume et al.	2019	Berthaume et al. (2019)	CT scan	Asia	18	8	10

### Regional and global rates

All 65 studies were included to investigate regional variability in prevalence rates, providing a sample of 21 626 knees and representing all regions and methods of data collection (results presented in Table 2 from Berthaume et al. 2019). Not all regions were equally represented, with studies per region ranging from 1 to 24 (Table 5, Fig. 5). The random intercept model was favoured over the random slope model (Appendix S3, weight = 1). There was significant variation in prevalence rates in region, year, and methodology of data collection (Table 5, Fig. 5). As before, prevalence rates are higher when data were collected with dissections compared with X‐rays and this increased with time. As only ~ 1% of the sample was collected using MRIs (*n* = 247 knees), and all MRI‐based data were collected relatively recently (Yu et al. 1996; De Maeseneer et al. 2001; Hedderwick et al. 2017), no estimates were given for MRI‐based prevalence rates.

**Table 5:** Median and 95% confidence interval fabella prevalence rates.

Region	Number of studies	Number of knees	1875		2018	
			Dissection	X‐ray	Dissection	X‐ray
Africa	3	350	9.63 (7.13–12.79)	4.07 (2.47–6.46)	22.37 (17.5–27.78)	10.29 (6.53–15.49)
Asia	24	4501	28.51 (26.17–30.95)	13.71 (9.47–19.04)	51.86 (49.64–54.12)	30.07 (22.17–38.8)
Europe	24	11 466	16.17 (15.03–17.38)	7.14 (4.86–10.11)	34.27 (31.61–37.01)	17.21 (12.07–23.52)
Middle East	1	1000	13.95 (11.47–16.81)	6.06 (3.97–9.04)	30.47 (26.88–34.23)	14.85 (10.23–20.8)
North America	7	3506	11.59 (10.11–13.23)	4.96 (3.32–7.22)	26.17 (23.71–28.77)	12.37 (8.54–17.26)
Oceania	3	164	28.21 (21.95–35.48)	13.51 (8.5–20.82)	51.5 (43.97–59.1)	29.7 (20.35–40.98)
South America	3	639	21.75 (17.62–26.44)	9.95 (7.15–13.49)	42.89 (37.58–48.25)	23.03 (17.58–29.02)
Worldwide	65	21 626	17.71 (11.26–27.33)	7.9 (4.26–14.15)	36.8 (25.71–50.39)	18.86 (10.76–30.45)

Prevalence rates were highest in Asia (30.07–51.86% in 2018), followed by Oceania (29.70–51.50% in 2018), with the lowest prevalence in Africa (10.29–22.37% in 2018) and North America (12.37–26.17% in 2018; Table 5, Fig. 5). Worldwide prevalence rates were calculated by assuming the average effect of country. In 2018, median prevalence rates are 36.80% and 18.86% for data collected through dissections and X‐rays, respectively.

## Discussion

**Figure 6:**
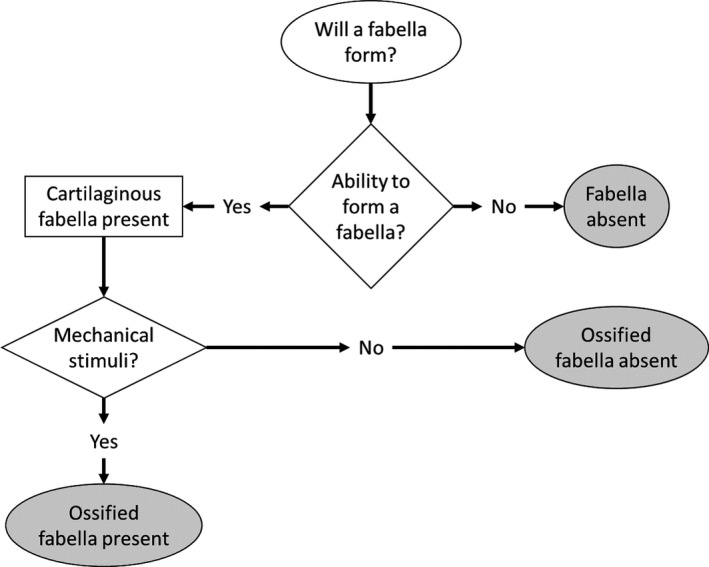
Flow chart depicting how fabellae form. Ability to form a fabella refers to the genetic component of fabella growth and development.

This meta‐analysis provides evidence that confirms some of the genetic and environmental explanations for variations in fabella prevalence rates, while bringing to light some new ones. We propose that, to grow an ossified fabella, individuals require both the ability to form a fabella and the mechanical stimuli necessary for fabella ossification (Fig. 6), where the ability to form a fabella is primarily genetically controlled and while fabella ossification is primarily environmentally controlled (Eyal et al. 2019).

Contrary to previous studies, we found sexual dimorphism in fabella prevalence rates. Fabellae have been, on average, 1.32–2.60% more common in men than women over the last 143 years, and 2.47–2.60% more common in men than women in 2018 (Fig. 1, Supporting Information Appendix S2). Sesamoid bones form in areas of high mechanical stimuli (e.g. friction, pressure, stress); without these stimuli, they do not ossify and/or become independent bones embedded in tendons (Eyal et al. 2015). Higher loads in the tendon of the lateral head of the gastrocnemius due to sexual dimorphism (i.e. the generally larger muscles, longer tibia in men, and more force in the tendon of the lateral head of the gastrocnemius) could produce more friction, tension, pressure, and stress in the tendon and explain higher prevalence rates in males. In both men and women, fabellae were more common in 2018 than in 1875, and studies that used dissection report higher prevalence rates than those that used X‐rays, possibly because it is easier to detect fabellae during dissection or because cartilaginous/less dense, ossified fabellae are being included in the fabella count (Table 1).

Previously, there was no consensus about the age of fabella ossification; these results offer the best evidence to answer this question. Prevalence rates increase ontogenetically, suggesting fabellae can ossify early in life – as early as 12 years of age (Pancoast, 1909; Ehara, 2014) – but may ossify later in life, potentially as late as 70 years old (Fig. 2). This is supported by Chung (1934), who found no cartilaginous fabellae in individuals over 60 years old, and that the ratio of ossified to cartilaginous fabellae increased with age. Unlike other skeletal elements, which ossify at predictable ages (Scheuer & Black, 2004), the fabella may ossify at a range of ages, stretching into adulthood. If fabellae ossify at an early age (e.g. during juvenility), there should be an increase in prevalence rates up until a certain age range, at which point prevalence rates should plateau; the only way an increase in prevalence rates could occur, is if at least some fabellae are ossifying later in life. If fabella formation, as with other sesamoid bones, occurs because of high mechanical loads, this could explain why some people develop them relatively early in life, whereas others develop them later. Some of the large variance in ossification prevalence rates could reflect population differences in activity level – populations that are more active (and hence have more mechanical stimuli) when young could have higher prevalence rates at younger ages compared with less active populations.

The apparent decrease in prevalence rates in the 71–80 and 80+ age ranges is likely a function of decreased sample size, and not because the fabella is resorbed with age, as there is no evidence suggesting the latter. As with the results for sexual dimorphism, prevalence rates are higher for each cohort with dissection‐based studies than with X‐ray studies (Figs 2 and S2).

Case reports show that ossified fabellae are found in both active and inactive individuals. For example, Zenteno et al. (2010) reported on a fabella in a 27‐year‐old female high‐performance Olympic level runner, and Kuur (1986) and Dashefsky (1977) reported on fabellae in 19‐ and 13‐year‐old male soccer players, supporting the idea that fabellae ossify due to mechanical loading (Dashefsky, 1977; Kuur, 1986; Zenteno et al. 2010). Conversely, the literature is saturated with case reports in older, presumably less active individuals (Laird, 1991; Ando et al. 2017), which appears to run contrary to fabella ossification under high loads. However, case reports generally only report on fabellae that are problematic, unless fabella prevalence was determined incidentally when investigating a problem with the knee. The patient's age when the fabella is discovered is then the maximum age of ossification for that individual, and in the case of elderly patients, it is possible the fabellae ossified earlier in life.

Taken together, the differences in prevalence rate due to sexual dimorphism and ontogeny support the idea that fabella ossification is a product of mechanical stimuli. The ability to form a fabella, however, appears to be primarily genetically controlled (see below).

During fetal growth and development, a highly genetically controlled process, bilateral, mediolateral symmetry is the default setting, and asymmetry only develops in the presence of additional inputs, such as environmental or further genetic signalling (Palmer, 2004). Evidence for genetic control over the ability to form a fabella is supported by Jin et al. (2017), who found bilateral, cartilaginous fabellae in 4/5 of the 15‐ to18‐week‐old fetuses examined (Jin et al. 2017). The higher percentage of bilateral cases of ossified fabellae in adolescents and adults further points towards a genetic basis for the ability to form a fabella.

Consistent with previous studies, fabellae are significantly more likely to be present bilaterally (72.94% of cases) than unilaterally (26.99% of cases; Fig. 3). Our results fit firmly within the range reported in prevalence rate studies, and are significantly lower than the ~ 80% commonly reported in the literature (95% CI for bilateral cases: 69.82–75.73%, Appendix S2) (Sutro et al. 1935; Pritchett, 1984; Dalip et al. 2018). When only one fabella is found, it is equally likely to be found in the right or left knee (*P* = 0.9992; Fig. 4). Interestingly, results in Appendix S3 imply there is no regional variation in the ratio of bilateral/unilateral cases, as the statistical models with regional variation performed worse than the model that did not include it. Similarly, method and year of data collection are both unimportant in predicting the percent of bilateral/unilateral cases.

If the ability to form a fabella were environmentally controlled, asymmetry should be the default case, and unilateral cases should be more common, particularly in fetuses. The antisymmetry (i.e. non‐directional asymmetry) in unilateral cases implies that the direction of asymmetry in unilateral cases is not inherited (Palmer, 2004), as genetic control in structural asymmetry generally manifests in directional asymmetry. If it were genetically controlled, ossified fabellae should be more common in one knee than the other.

Genetic bases for fabella formation are further supported by regional variation in prevalence rates, suggesting populations of certain genetic ancestries are more/less likely to form fabellae than others. Literature on fabella prevalence rates often states fabellae are more common in Asian, and in particular Japanese (Hessen, 1946), populations, and rarer in populations of non‐Asian ancestry (Chew et al. 2014). However, studies rarely quantitatively compare prevalence rates between populations, and when they do, it is generally with relatively small sample sizes (Miaśkiewicz & Partyka, 1984), not taking advantage of the literature. As such, the relatively high prevalence rates in Oceania populations have generally gone unnoticed.

This is the first study to quantify variation in fabella prevalence rates across different populations, and to provide global prevalence rates. Fabellae are the most common in populations from Asia, followed by those from Oceania, South America, Europe, the Middle East, North America, and Africa. It should be noted that the populations used to create these regional groupings may or may not consist of genetically homogeneous and distinct populations. Further, we assume the individuals included in each study have an ethnic heritage corresponding with the country in which the study was conducted – this is, of course, likely not true for some studies. In these situations, these data are clinically useful for determining modern prevalence rates.

It is possible the higher prevalence in certain populations is correlated to some morphological characteristic(s) of those populations. For example, features of femoral/tibial shape more prevalent in Asian/Oceania populations may change the mechanical loading at the knee, creating a mechanical stimulus that promotes fabella formation. However, we cannot assess these arguments properly as we lack morphological data from the individuals used in these studies. The study by Jin et al. (2017) also showed that prevalence rates from cartilaginous fabellae are high in Japanese populations *in utero*, implying that femoral/tibial morphological parameters, particularly ones affected by ontogeny, are not responsible for cartilaginous fabella formation, although they may play a role in fabella ossification.

We are hesitant to hypothesize about any morphological changes in the femur/tibia that may be responsible for variation in global fabella prevalence rates, as this would require a morphological cline with Asians at one extreme and Africans at the other. It is not possible to conclude from these data whether fabella presence is determined directly by genetics, but rather these data imply there is a genetic component correlated to ossified fabella presence. Whether that is a (set of) gene(s) signalling for fabellae to form, directly, or a (set of) gene(s) that create a morphological environment that makes it more likely for ossified fabellae to form, we cannot say.

In 2018, the global prevalence rates for fabellae were 36.80% (95% CI 25.71–50.39%) for dissection‐based studies, and 18.86% (95% CI 10.76–30.45%) for X‐ray‐based studies. A global prevalence rate of 36.80% is higher than the 10–30% commonly reported in the literature (Duncan & Dahm, 2003; Dalip et al. 2018).

Taken together, these data suggest that the ability to form a fabella is primarily genetically controlled. This conclusion is most strongly supported by the high percentage of bilateral cases and the regional variation in prevalence rates. The gene(s) and genetic pathways responsible for the presence/absence of fabellae are unknown, but as their presence/absence is correlated to the presence/absence of the os peroneum, it is possible these sesamoid bones operate under the same genetic control (Sarin et al. 1999).

Fabella ossification, however, may be controlled by environmental, functional factors, such as mechanical stimuli. This conclusion is most strongly supported by the sexual dimorphism and ontogenetic data, which show a higher prevalence rate in males, an increasing prevalence rate with age, and antisymmetry within unilateral cases. The functional pathways responsible for fabella ossification are currently unknown. Occasional, high forces acting on the tendon of the lateral head of the gastrocnemius would increase the maximum mechanical stimuli experienced by the tendon and may trigger fabella ossification. In the same way, repetitive, lower forces, like those experienced during walking, could produce constant, low‐level mechanical stimuli, which is known to trigger bone modelling/remodelling in a manner similar to high, occasional loads (Ruff et al. 2006).

Like the patella, it is possible the fabella provides a functional advantage when present, increasing the lever arm of the muscle when the leg is straight (Eyal et al. 2015). When the leg is bent, however, and the fabella is no longer pressed against the posterior surface of the lateral condyle, it is unlikely to confer such a mechanical advantage. It is also possible that the fabella, and thereby fabellofibular ligament, offers some type of advantage in redirecting some of the forces produced by the gastrocnemius from the femur to the fibula. However, if these exist, these functional advantages are unlikely to be related to the mechanical stimuli that cause ossification.

Although this study provides invaluable data about the fabella, there are some limitations. First, the use of studies from such a variety of countries, languages, and spanning such a broad range of time may mean methods employed for fabella detection may differ, and the data from these studies are not directly comparable. Secondly, the unequal sample of knees per study, studies per year, and studies per country may have led to a bias in the results, particularly in estimating worldwide fabella prevalence. Lastly, the relatively low number of studies included in the ontogenetic analysis may have skewed the results.

## Conclusions

Fabellae are ~ 3.5 times more common in 2018 than they were in 1918, and are correlated to a number of biological questions, ranging from medicine to evolution, making it pertinent to understand variation in fabellae prevalence rates, and how prevalence rates are affected by genetic and environmental factors. For the first time, we are able to prove that sexual dimorphism in fabella prevalence exists, and fabellae are more common in men than women. Also for the first time, we show that fabella prevalence rate increases with age, implying fabellae may ossify early in life (as early as 12 years old) or later (as late as 70 years old). Consistent with the literature, the majority of cases where a fabella is present are bilateral, and within unilateral cases, fabellae are as likely to be present in the right as in the left knee. And finally, there is marked regional variation in fabella prevalence rates, with fabellae being more common in Asian, Oceania, and South American populations than in European, Middle Eastern, North American, and African ones. On average, 36.80% of knees, worldwide, have a fabella.

## Author contributions

M.A.B. and A.M.J.B. conceived of and designed the project. M.A.B. acquired/analysed the data and performed statistical analyses. M.A.B. and A.M.J.B. wrote/edited the manuscript and approved its final version.

## Supporting information


**Appendix S1** Raw data.xlsx: Raw data used for the meta‐analysis.

**Appendix S2** Median prev rates and CIs.xlsx: Median prevalence rates and confidence intervals, calculated from the posterior distributions of the models with the lowest WAIC.

**Appendix S3** WAIC results for our statistical models.

**Appendix S4** Posterior distributions for model parameters.docx: statistical models used to interpret our results and their posterior distributions.
