# Supplementary material for: Human biological variation in sesamoid bone prevalence: the curious case of the fabella
Source: J Anat. 2019 Oct 17;236(2):228–42. doi: 10.1111/joa.13091 (PMC6956444; doi:10.1111/joa.13091)
Supplement: Supplementary file 3 — Appendix S3 WAIC results for our statistical models. [file JOA-236-228-s003.docx]

WAIC results. X’s and O’s indicate inclusion and exclusion of the parameter from the model, respectively. pWAIC is the effective number of parameters, dWAIC the difference in WAIC scores between each score and the top weight model, weight the probability that that is the best model, SE the standard error in WAIC calculation, and dSE the standard error of the difference in WAIC between each model and the top ranked model (McElreath, 2016). The two models for region included all parameters.

|  | Region | Method | Year | WAIC | pWAIC | dWAIC | weight | SE | dSE |
| --- | --- | --- | --- | --- | --- | --- | --- | --- | --- |
| Sexual Dimorphism | X | X | X | 7992.5 | 5.8 | 0 | 1 | 96.85 | NA |
|  | O | O | X | 8027.5 | 5.0 | 35.0 | 0 | 96.66 | 11.75 |
|  | X | X | O | 8093.2 | 3.0 | 100.7 | 0 | 96.18 | 20.56 |
|  | O | O | O | 8149.9 | 2.0 | 157.3 | 0 | 95.88 | 25.69 |
|  |  |  |  |  |  |  |  |  |  |
| Age | X | X | O | 4142.5 | 11.0 | 0 | 0.53 | 72.47 | NA |
|  | X | X | X | 4142.8 | 11.8 | 0.2 | 0.47 | 72.48 | 2.05 |
|  | O | O | X | 4198.2 | 9.0 | 55.6 | 0 | 71.89 | 15.22 |
|  | O | O | O | 4198.4 | 7.9 | 55.8 | 0 | 71.82 | 15.25 |
|  |  |  |  |  |  |  |  |  |  |
| Bilateral/ unilateral | O | O | O | 2103.7 | 2.0 | 0 | 0.63 | 37.56 | NA |
|  | X | O | O | 2106 | 3.1 | 2.1 | 0.22 | 37.58 | 0.05 |
|  | X | X | O | 2107.3 | 3.8 | 3.6 | 0.10 | 37.63 | 0.08 |
|  | X | X | X | 2109.2 | 4.8 | 5.5 | 0.04 | 37.71 | 0.17 |
|  |  |  |  |  |  |  |  |  |  |
| Sidedness | O | O | O | 575.2 | 2.0 | 0 | 0.60 | 0.01 | NA |
|  | O | O | X | 577.0 | 2.9 | 1.8 | 0.25 | 0.08 | 0.09 |
|  | X | X | O | 578.7 | 3.8 | 3.5 | 0.11 | 0.07 | 0.07 |
|  | X | X | X | 580.2 | 4.5 | 5.0 | 0.05 | 0.10 | 0.10 |
|  |  |  |  |  |  |  |  |  |  |
| Region | random intercepts | | | 20320.2 | 10.7 | 0.0 | 1 | 168.44 | NA |
|  | random slopes | | | 20503.4 | 10.3 | 183.2 | 0 | 167.32 | 27.11 |
