# Supplementary material for: Human biological variation in sesamoid bone prevalence: the curious case of the fabella
Source: J Anat. 2019 Oct 17;236(2):228–42. doi: 10.1111/joa.13091 (PMC6956444; doi:10.1111/joa.13091)
Supplement: Supplementary file 4 — Appendix S4 Posterior distributions for model parameters.docx: statistical models used to interpret our results and their posterior distributions. [file JOA-236-228-s004.docx]

**Supplementary information: posterior distributions**

*Sexual Dimophism*

Model: sex.diff

sex.diff <- map2stan(

alist(

Fabellas ~ dbinom(Extrem,p),

logit(p)<-a + a_coun[Country2] + a_meth[Method2] +by*Year + b*Sex,

a ~ dnorm(0,10),

a_coun[Country2] ~ dnorm(0,sigma_coun2),

sigma_coun2 ~ dcauchy(0,1),

a_meth[Method2] ~ dnorm(0, sigma_meth2),

sigma_meth2 ~ dcauchy(0,1),

by ~ dnorm(0,50),

b ~ dnorm(0,50)

),

data=data_sex, chains = 4, iter = 10000, warmup = 1000

)


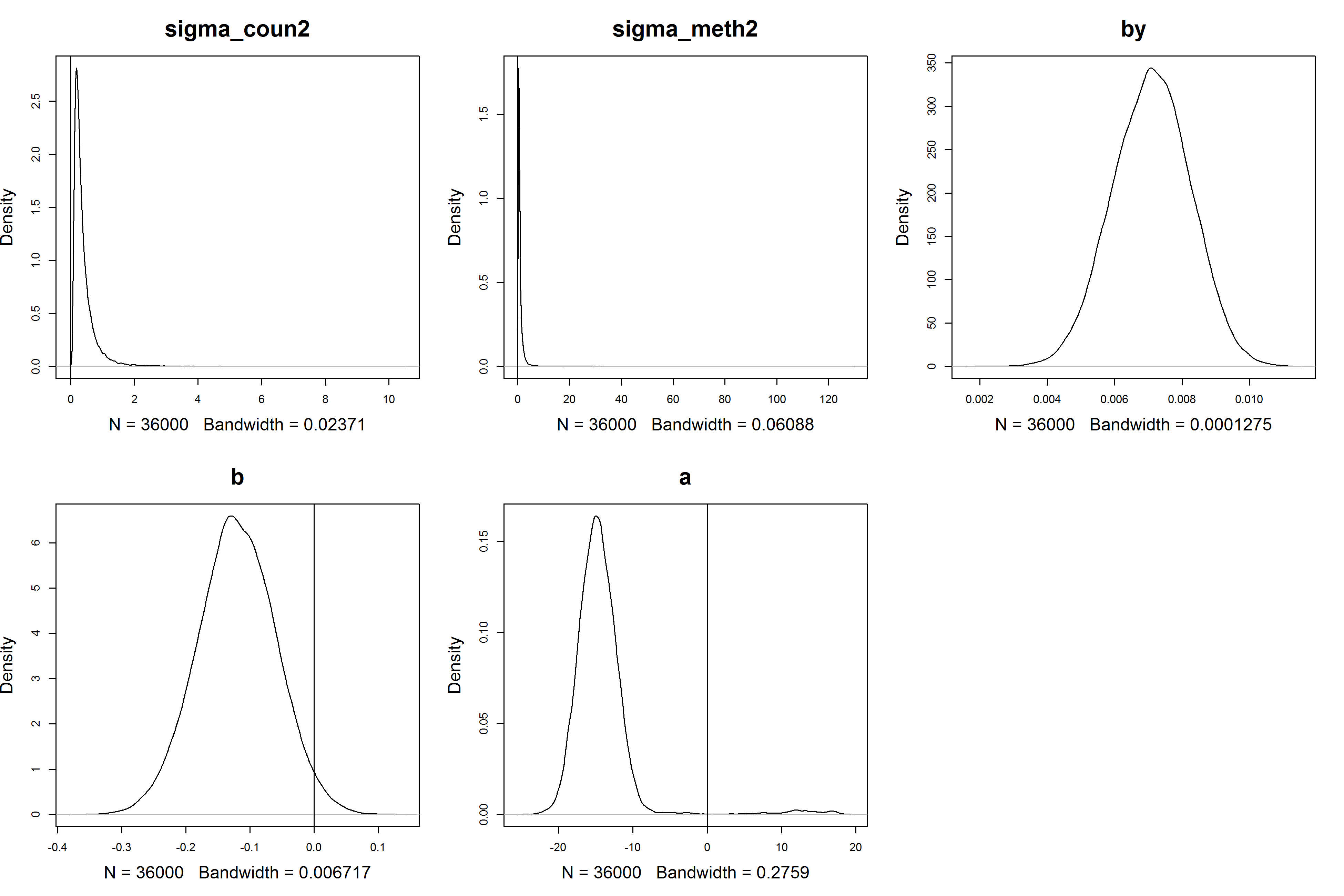


Figure 1: Posterior distributions for sex.diff model.

*Ontogeny*

Model: age

age <- map2stan(

alist(

Fabellas ~ dbinom(Sample,p),

logit(p)<-a + a_coun[Country2] + a_meth[Method2] + a_age[Age2],

a_coun[Country2] ~ dnorm(0, sigma_coun2),

sigma_coun2 ~ dcauchy(0,1),

a_meth[Method2] ~ dnorm(0, sigma_meth2),

sigma_meth2 ~ dcauchy(0,1),

a_age[Age2] ~ dnorm(0, sigma_age2),

sigma_age2 ~ dcauchy(0,1),

a ~ dnorm(0,10)

),

data=data, chains = 4, iter = 10000, warmup = 2000

)


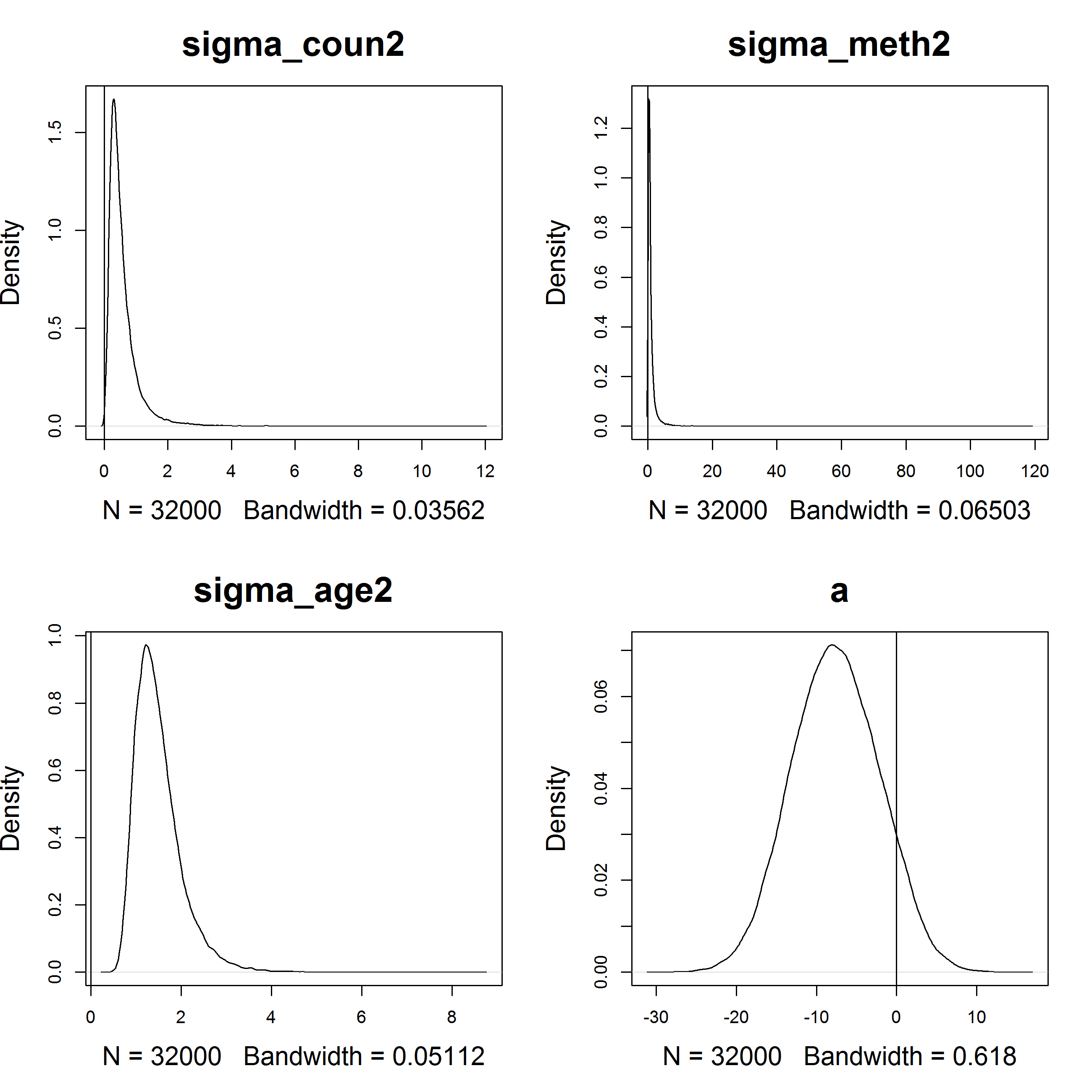


Figure 2: Posterior distributions for age model.

*Bilateral vs. unilateral*

Model: bi.uni

bi.uni <- map2stan(

alist(

Num_bu ~ dbinom(Samp_bu,p),

logit(p)<-a + b*bi_uni,

a ~ dnorm(0,10),

b ~ dnorm(0,50)

),

data=data_bu, chains = 4, iter = 10000, warmup = 1000

)


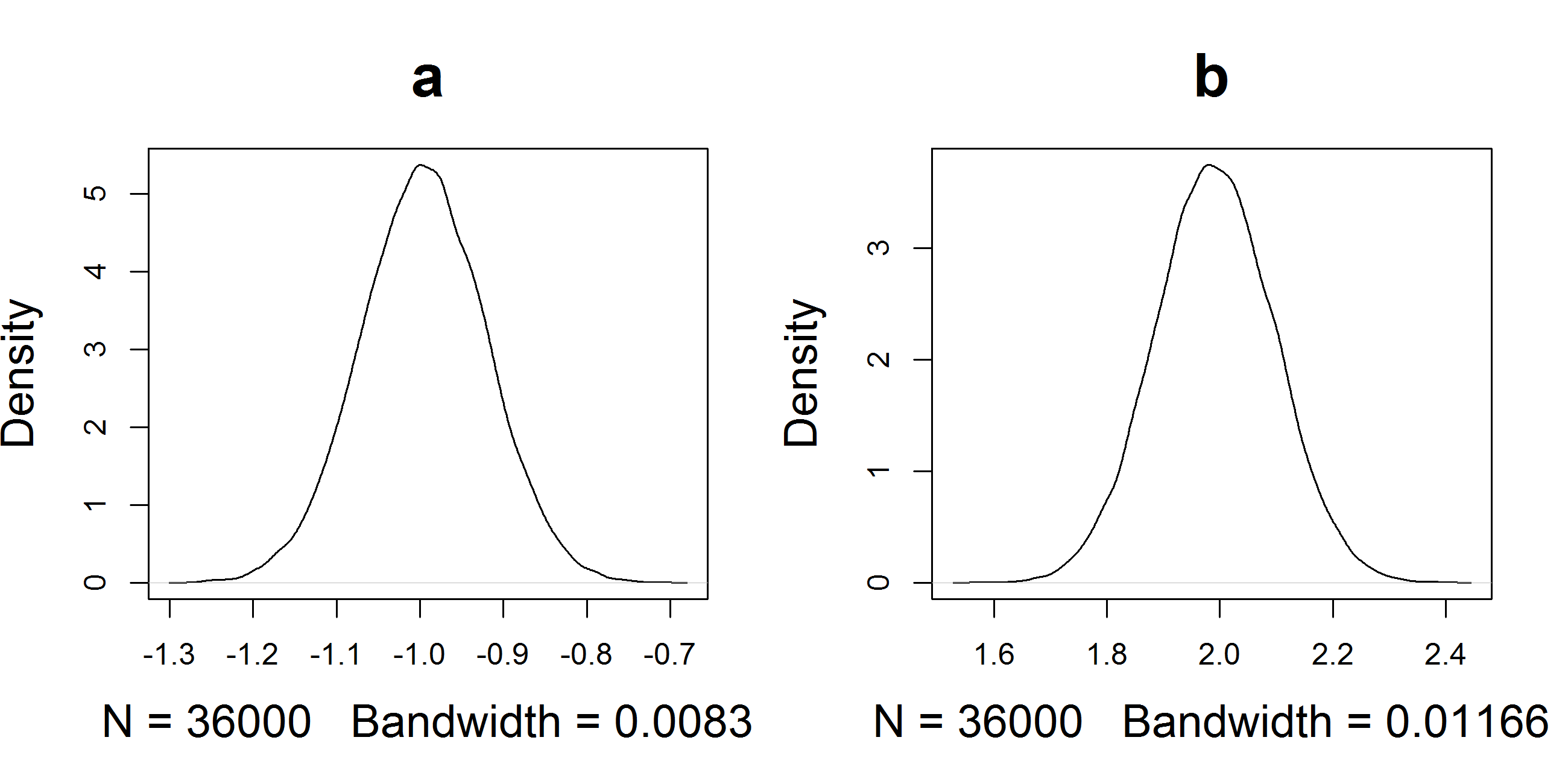


Figure 3: Posterior distributions for bi.uni model.

*Sidedness*

Model: m.rl

m.rl <- map2stan(

alist(

Num_RL ~ dbinom(Samp_RL,p),

logit(p)<-a + by*Year + b*R_L,

a ~ dnorm(0,10),

by ~ dnorm(0,50),

b ~ dnorm(0,50)

),

data=data_rl, chains = 4, iter = 10000, warmup = 1000

)


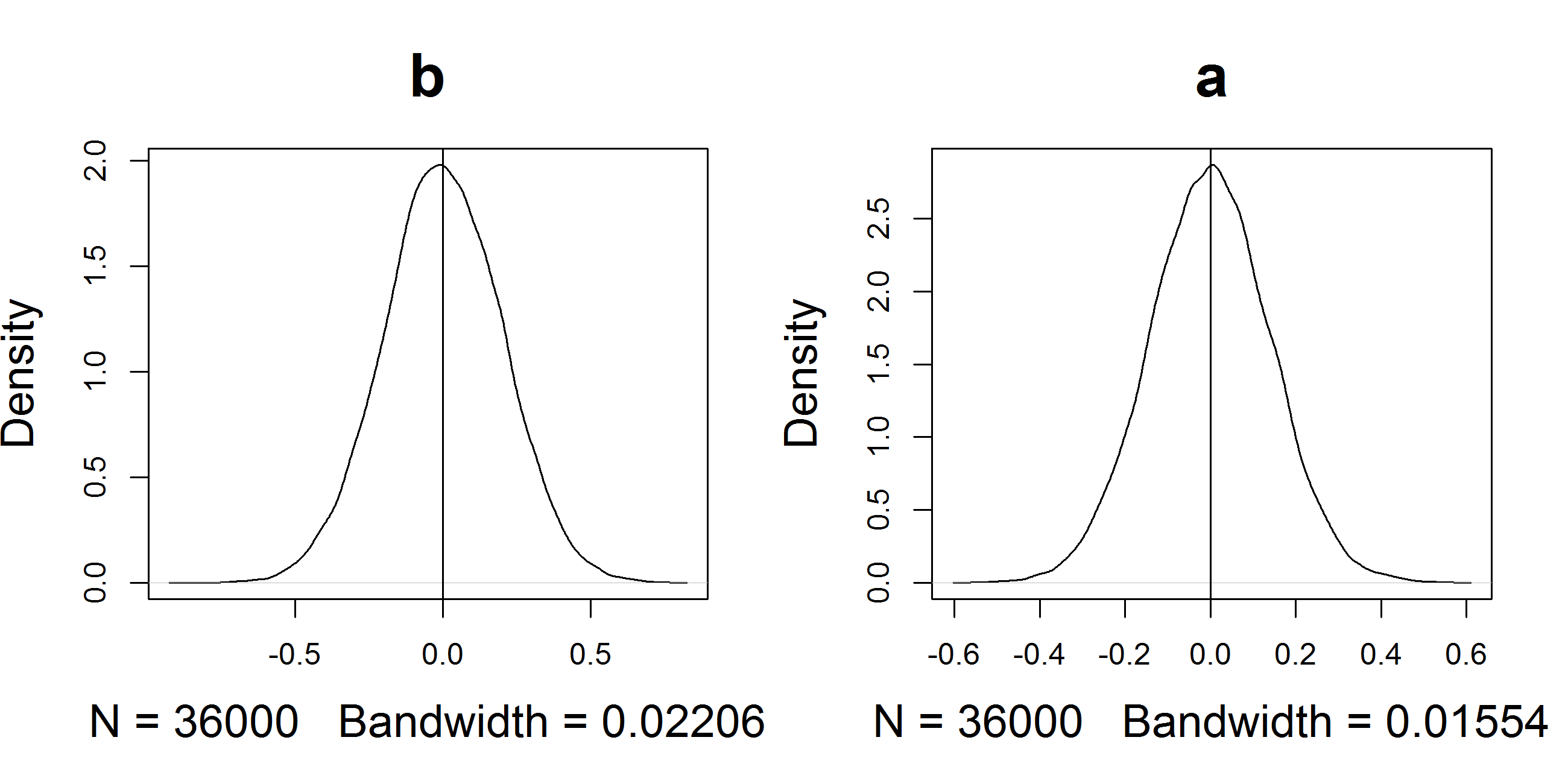


Figure 4: Posterior distributions for m.rl model.

*Regional*

Model: prev.rate

prev.rate <- map2stan(

alist(

Number_of_fabellas ~ dbinom(Number_of_knees,p),

logit(p)<- a + a_coun[Country2] + a_meth[Method2] + by*Year,

a_coun[Country2] ~ dnorm(0, sigma_coun2),

sigma_coun2 ~ dcauchy(0,1),

a_meth[Method2] ~ dnorm(0, sigma_meth2),

sigma_meth2 ~ dcauchy(0,1),

a ~ dnorm(0,10),

by ~ dnorm(0,10)

),

data=data, chains = 4, iter = 10000, warmup = 2000

)


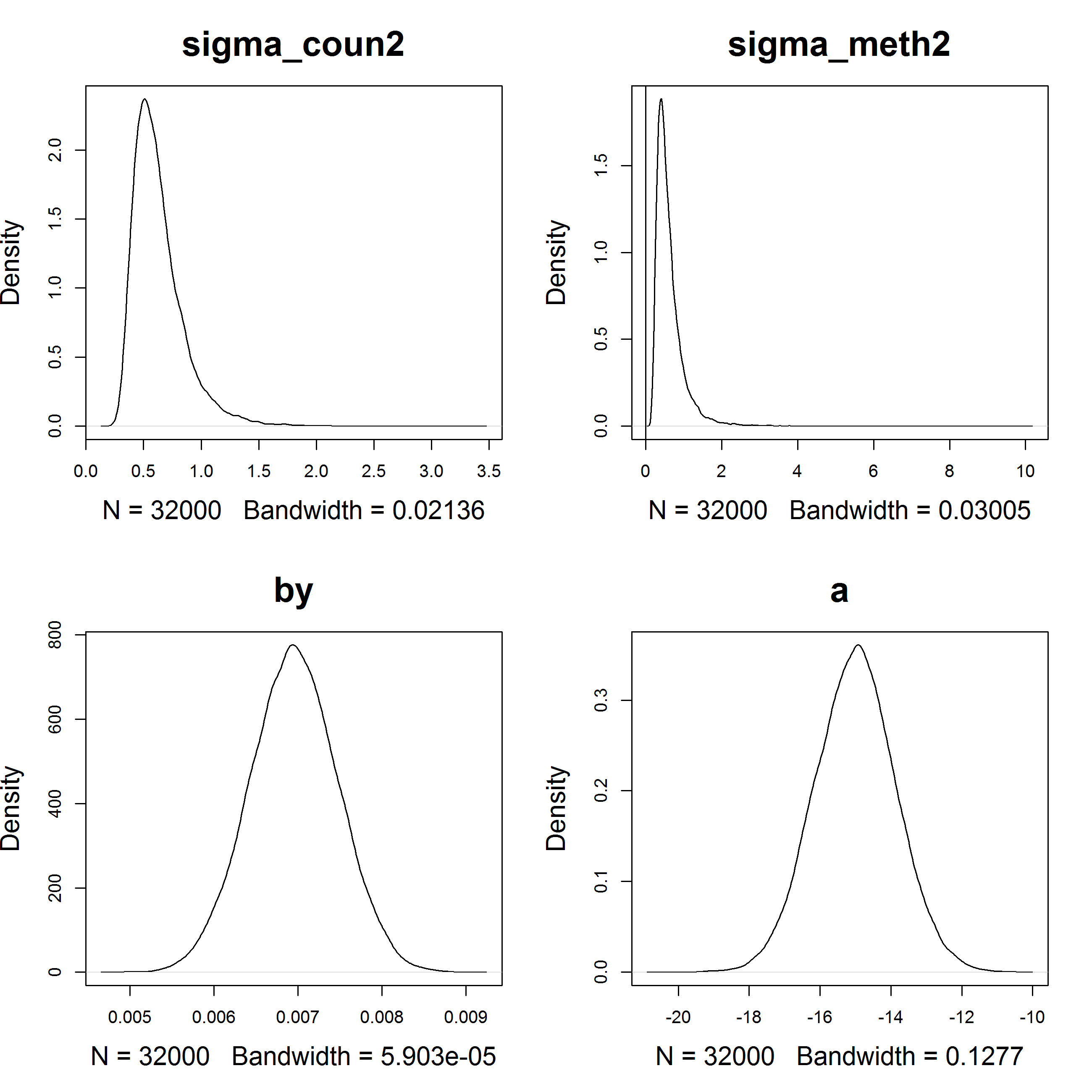


Figure 5: Figure 4: Posterior distributions for prev.rate model.
